# Guillain–Barre Syndrome following lower segment caesarean section under spinal anesthesia: A case report

**DOI:** 10.1002/ccr3.6427

**Published:** 2022-10-11

**Authors:** Prajjwol Luitel, Devansh Upadhyay, Nischal Neupane, Sujan Paudel, Prashant Gyawali, Bikram Prasad Gajurel, Ragesh Karn, Reema Rajbhandari, Niraj Gautam, Ashish Shrestha, Rajeev Ojha

**Affiliations:** ^1^ Maharajgunj Medical Campus Tribhuvan University Institute of Medicine Kathmandu Nepal; ^2^ Department of Neurology Tribhuvan University Institute of Medicine Kathmandu Nepal

**Keywords:** Guillain–Barre Syndrome, lower segment caesarean section, postpartum, surgery

## Abstract

Symptoms of Guillain–Barre Syndrome (GBS) may be mistaken for typical puerperal changes, delaying diagnosis. Surgery and anesthesia may be triggers for GBS with an overall increase in pro‐inflammatory cytokines in the postpartum period. We report a unique case of GBS in the postpartum period who made a good recovery with supportive measures.

## Abstract 2

Guillain–Barre Syndrome (GBS) symptoms may be misinterpreted as usual puerperal changes. Prompt diagnosis and management are crucial to minimize complications. We report a case wherein we managed a post‐surgical GBS with conservative agents resulting full recovery.

## 1 INTRODUCTION

Guillain–Barre Syndrome (GBS) is an acquired, monophasic, acute, or subacute onset of bilateral, symmetric weakening of varied degrees of limbs, with decreased or absent reflexes.^1^, ^2^ GBS can complicate any trimesters of pregnancy and postpartum period but is more common in third trimester and first 2 weeks postpartum.^3^, ^4^ After careful analysis of the clinical history, cerebrospinal fluid testing, and electrodiagnostic tests, the diagnosis is made. Neuroimaging may be necessary in some circumstances to rule out differentials. Because the early non‐specific symptoms may mimic changes in pregnancy, diagnosis is typical delayed during pregnancy or the immediate postpartum period. GBS should be considered in any pregnant woman who is experiencing muscle weakness, overall malaise, tingling in her fingers, or breathing problems.^5^ We report a case of GBS in the immediate postpartum after a surgical delivery under spinal anesthesia who was managed conservatively and recovered completely.

## 2 CASE PRESENTATION

A 27‐year‐old primipara underwent emergency lower segment caesarean section (LSCS) under spinal anesthesia for fetal distress at 38 weeks of gestation. She was discharged on fifth postoperative day and the recovery was uneventful except numbness and tingling in her lower limbs which continued for 1 week.

On the 22nd postoperative day, she was readmitted (early March of 2021) with history of progressive weakness in bilateral upper and lower limbs for 2 weeks. From 8th postoperative day, she had insidious onset, symmetrical weakness in bilateral upper and lower limbs which progressed in distal to proximal pattern. During initial first 2 weeks (8th–22ndth postoperative day) of weakness, she was able to do her daily chores with support of her family members but later completely lost the ability to stand and support. She also complained of burning and tingling sensation in her lower limbs, associated with reduced sensation to touch. However, there was no history of aspiration, nasal regurgitation, or intonation, swallowing difficulty, headache, photophobia, double vision, abnormal body movements, loss of consciousness, and bowel or bladder incontinence. She had no history suggestive of gastrointestinal or respiratory illness prior to the onset of her symptoms, similar illnesses in the past, no significant familial, medical, surgical, traumatic, and vaccination history.

On examination, resting pulse was 110 beats per minute, regular rhythm, blood pressure 100/80 mm of Mercury, respiratory rate 20 cycles per minute and oxygen saturation 97% in room condition. Higher mental functions were intact. Cranial nerves were intact, with no facial deviation or swallowing difficulty. Muscle bulk and tone across joints were normal. Power of shoulder abduction, adduction, elbow flexion, extension, and wrist flexion, extension were 5/5, hand grip strength was 80%, hip abduction, adduction 2/5, hip flexion, extension 2/5, knee flexion and extension 0/5, and toe flexion, extension 0/5. Biceps reflex was 2+, triceps reflex was 2+. Knee reflex and ankle reflex were absent. Plantar reflex was downgoing bilaterally. Sensations to touch, pin prick, and vibrations were intact in both upper and lower limbs.

Routine laboratory parameters were within normal limits. Cerebrospinal Fluid (CSF) revealed albumin‐cytologic dissociation with protein 71.6 mg/dl, total leucocyte count <5 cells all being monomorphs, sugar 64 mg/dl, Adenosine deaminase 4.8 U/L (normal 0–30 U/L), no Red Blood Cells, and no growth in CSF culture after 48 h of incubation. Nerve Conduction tests (NCT) revealed generalized severe sensory polyneuropathy, axonopathy of bilateral posterior tibial nerve. According to these findings, acute motor‐sensory axonal neuropathy variant of GBS was considered. Computed tomography (CT) scan Head and Magnetic Resonance Imaging (MRI) whole cervical spine was normal.

Absence of upper motor signs, insignificant MRI findings, symmetrical weakness with presence of sensory symptoms and NCT findings helped to rule out possibility of transverse myelitis, lumbar epidural hematoma, poliomyelitis, respectively, and finally diagnosis of GBS was made. Her condition fell in Level 1 of Brighton criteria and grade 4 Hughes Functional Grading Scale (HFGS). Patient was counseled for treatment of intravenous immunoglobulin which is quite expensive in Nepal (about 3000 USD for patient of 65 kg body weight). Due to low economic condition, she was managed conservatively with physiotherapy, calcium and multivitamin supplements. After admission, patient's symptoms were static and did not involve respiratory difficulties. There was progressive improvement in her weakness of upper and lower limbs. On discharge after 2 weeks of hospital stay, her power improved to 4+/5 in bilateral lower limbs. However, knee and ankle reflex were still absent. There was mild tingling sensation in bilateral lower limbs, and no abnormal sensations in upper limbs. In 1‐month follow‐up, patient power completely improved with no sensory symptoms (Tables 1 and 2).

**TABLE 1:** Nerve conduction study: Motor

Nerve	Latency (ms)		Amplitude (mv)		NCV (m/s)	F‐Min (ms)
	D	P	D	P		
Rt. CPN	3.62	9.94	5.83	2.49	47.47	50.63
Lt. CPN	2.94	9.81	10.11	12.25	43.67	51.12
Rt. PTN	3.00	NS	4.23	NS	–	–
Lt. PTN	3.62	NS	7.75	NS	–	–
Rt. Median	2.69	6.81	26.74	25.27	50.97	25.00
Rt. Ulnar	1.87	5.19	21.62	22.22	63.25	23.01
Lt. Median	3.42	7.24	18.22	17.26	51.56	26.14
Lt. Ulnar	3.12	6.27	16.54	16.24	53.88	24.65

Abbreviations: CPN, Common Peroneal Nerve; D, distal; Lt, left; NCV, nerve conduction velocity; NS, not stimulated; P, proximal; PTN, Posterior Tibial Nerve; Rt, right.

**TABLE 2:** Nerve conduction study: Sensory

Nerve	Latency (ms)	Amplitude (μv)	NCV (m/s)
Rt. Sural	1.37	3.35	72.99
Lt. Sural	NS	NS	NS
Rt. Median	2.48	9.76	52.42
Rt. Ulnar	NS	NS	NS
Lt. Median	NS	NS	NS
Lt. Ulnar	NS	NS	NS

Abbreviations: Lt, left; NCV, nerve conduction velocity, NS, not stimulated; Rt, right.

## 3 DISCUSSION

The incidence of GBS ranges from 0.4 to 4 cases/1000 across all ages.^6^ Post‐surgical GBS is defined as occurrence of clinical features suggestive of GBS within 6 weeks of surgery in absence of major triggers.^7^ Most common surgeries accounting for post‐surgical GBS are orthopedic surgery, neurosurgery, gastrosurgery, and cardiac surgery. Across different studies, post‐surgical GBS has accounted for 4.5%–9.1% of total cases of GBS.^8^, ^9^

Guillain–Barre Syndrome can complicate any trimesters of pregnancy and postpartum period but is more common in third trimester and first 2 weeks postpartum.^3^, ^4^ The restoration of cellular immunity, which had been adaptively repressed throughout pregnancy, is considered to cause or aggravate GBS during the postpartum period.^10^, ^11^ Besides it, etiology of post‐surgical GBS are release of antigens during surgery sensitizing immune system towards development of autoimmunity for peripheral nerve antigens, cell‐mediated immunosuppression, neuroendocrine axis activation, transfusion possibly causing sub‐clinical infection leading to cross‐reactive antibodies, inflammation resulting from damage to blood vessels, spinal cord ischemia, hemorrhage, and direct nerve injury.^8^, ^9^, ^12^, ^13^, ^14^, ^15^

In our case, a post‐surgical GBS was confirmed by clinical symptoms, characteristic disease progression and improvement, CSF findings, electrodiagnostic testing and ruling out focal lesions by neuroimaging and history of onset of symptoms within 6 weeks of surgery. But still, GBS symptoms might be misinterpreted as usual puerperal changes, delaying diagnosis.^16^ Another differential could be post‐surgery myelitis, as the patient had chief clinical symptoms in lower limbs only. However, nerve conduction study showed sensory axonal neuropathy of upper limbs as well. Further, there was no sensory level, bowel and bladder functions were normal, and no findings suggestive of upper motor lesions.

Post‐surgical GBS patients have high HFGS at admission, at nadir and at discharge and a longer duration of hospital stay.^9^ Our patient being completely bedridden during the presentation was also in Grade 4 HFGS. Median duration from surgery to onset of symptom is 15 days,^8^ but it has been documented to occur as early as 5 h after surgery.^17^ It was 7 days in our particular case. Post‐surgical GBS are commonly of AMAN or ASMAN variants, electrophysiological study revealing predominant damage in axons.^18^, ^19^ Our patient had the ASMAN subtype of GBS. This axonal pattern leads to bad prognosis compared with non‐surgical GBS patients.^18^ The incidence of acute respiratory failure and mechanical ventilation in post‐surgical GBS patients is significantly higher than non‐surgical GBS patients, thus requiring frequent evaluations of pulmonary function.^18^, ^20^ However, our patient did not deteriorate further to involve bulbar or respiratory function.

## 4 CONCLUSION

A high index of suspicion of GBS is paramount since delay in diagnosis is common in pregnancy or early postpartum period because the initial non‐specific symptoms may resemble changes in the pregnancy.

## AUTHOR CONTRIBUTIONS

PL and DU involved in writing the manuscript, collection of case information, manuscript revision. RO involved in writing the manuscript concept, collection of case information, manuscript revision. NN, DU, SP, and PG participated in preparing a literature review and interpretation of clinical findings. BPG, RK, RR, NG, and AS involved in patient care team and collection of case information. All authors approved the final version.

## CONFLICT OF INTEREST

The authors declare they have no conflicts of interest.

## ETHICAL APPROVAL

Case reports are waived from ethical approval in our institution. I attest that my article submitted to Clinical Case Reports Journal. This report has not been published in its entirety or in part anywhere else. The manuscript is not currently being considered for publication in another journal. I was personally and actively involved in the substantive effort that resulted in the revised manuscript, and they will be jointly and individually accountable for its content.

## CONSENT

Written informed consent for the publication of this case report was obtained from the patient.

## Data Availability

The data that support the findings of this study are available on request from the corresponding author. The data are not publicly available due to privacy or ethical restrictions.
